# Extraction of Bioactive Compounds From *Cannabis sativa* L. Flowers and/or Leaves Using Deep Eutectic Solvents

**DOI:** 10.3389/fnut.2022.892314

**Published:** 2022-05-02

**Authors:** Francisco J. Tiago, Alexandre Paiva, Ana A. Matias, Ana Rita C. Duarte

**Affiliations:** ^1^LAQV/REQUIMTE, Chemistry Department, NOVA School of Science and Technology, Monte de Caparica, Portugal; ^2^DES Solutio, Torres Vedras, Portugal; ^3^Clever Leaves Portugal, Lisbon, Portugal

**Keywords:** *Cannabis sativa*, green extraction, deep eutectic solvents, cannabinoids, plant extracts

## Abstract

The increasing demand for medical cannabis urges the development of new and effective methods for the extraction of phytocannabinoids. Deep eutectic solvents (DESs) are an alternative to the use of hazardous organic solvents typically used in the industry. In this study, hydrophilic and hydrophobic DESs were developed based on terpenes, sugars, and natural organic acids as green extraction media for the extraction of cannabis bioactive compounds. The factors influencing the extraction of bioactive components, such as the type of DESs and extraction time, were investigated. Initial screening in hemp showed that the DES composed of Men: Lau (a 2:1-M ratio) had a greater extraction efficiency of cannabidiol (CBD) and cannabidiolic acid (CBDA) (11.07 ± 0.37 mg/g) of all the tested DESs and higher than ethanol. Besides having a higher or equivalent extraction yield as the organic solvents tested, DESs showed to be more selective, extracting fewer impurities, such as chlorophyll and waxes. These results, coupled with the non-toxic, biodegradable, low-cost, and environmentally friendly characteristics of DESs, provide strong evidence that DESs represent a better alternative to organic solvents.

## Introduction

Cannabis is one of the world’s oldest cultivated and widely distributed plants (1). Historically, cannabis plants were cultivated for two main applications: as a medicine in Asia, mainly in India, and for fibers, which were used for the production of textiles, especially in the western world (2–4). The numerous years of cultivation and selection lead to the production of a wide range of varieties and hybridizations. Due to that, cannabis taxonomy is still an ongoing debate, and the classification of the different varieties can be based on plant phytochemistry. The chemotaxonomy of the plants is based on the ratio between Δ9- tetrahydrocannabinol (THC) and the cannabidiol (CBD), since the ratio between these molecules remains constant throughout the plant’s life. Different chemotypes range from plants that contain Δ9-THC as the predominant cannabinoid to plants that contain CBD as the predominant cannabinoid and to a variety of mixtures of the two (5). Based on this classification, it is possible to differentiate both, hemp, the fiber-type plants and marijuana or medical cannabis, referring to drug-type plants (6). European industrial hemp has 0.2–0.3% THC levels and higher levels of CBD (Type III), while drug-type contains 5–0% or more THC (Type I or Type II).

With the current changes in cannabis policies and new legislation for its medical use and cultivation, in the last 20 years, cannabis has been witnessing a revival, and, nowadays, domesticated forms of cannabis are spread and cultivated all over the world, exclusively for industrial purposes (5). These political changes led to the opening of a new sector of business due to the industrial and therapeutic potential of cannabis (7, 8).

Cannabis is a psychoactive plant that contains more than 500 different chemical compounds, of which cannabinoids are the main constituents (9, 10). These molecules are produced through the secondary metabolism, and their concentration varies between the different subspecies, age, harvesting time, and growing conditions (9). Cannabinoids, also referred to as phytocannabinoids, are a group of C21, or C22, considering the carboxylated forms, terpenophenolic compounds mainly produced in cannabis (9, 10). They are present in other plant genus, such as *Radula* and *Helichrysum*, but the knowledge of these sources is still very limited (1). In cannabis, these compounds are produced and accumulated in the glandular trichomes located in the aerial parts of the plant and are present in higher density on the female flowers (11).

Biosynthesis of cannabinoids happens along with cannabis secondary metabolism and starts with the bond between geranyl pyrophosphate (GPP) and olivetolic acid, creating cannabigerolic acid (CBGA). From this precursor, specific enzymes derivate other cannabinoids acids as cannabidiolic acid (CBDA) and Δ9-tetrahydrocannabinol acid (THCA) that can be converted non-enzymatically into their decarboxylated forms, cannabidiol (CBD), and Δ9-tetrahydrocannabinol (THC), respectively, either by light or heat, while in storage or when combusted (9). The most well-known conversion is from the not-psychoactive Δ9-THCA to the psychoactive compound Δ9-THC by smoking, a process that makes cannabis inflorescences a drug-related substance. Δ9-THC can be then oxidized to form cannabinol (CBN). Because THC and CBD are the major cannabinoids present in cannabis, they are the most studied and interesting compounds of the class (12).

Traditionally, the extraction of cannabinoids is performed using organic solvents, including hydrocarbons (e.g., hexane) and alcohols (e.g., ethanol, methanol). This method of extraction is cheap, easy to operate, and does not require sophisticated equipment; however, the solvents used are flammable, toxic, and non-biodegradable, risking human health, besides having a huge environmental impact (13). Extraction using these solvents can be efficient but depending on the final product, can impact regulation, and require additional testing. For instance, residual solvent is strictly regulated and must be defined for medicines under good manufacturing practice. These solvents due to their toxicity, environmental risk, and flammability are less desirable for large scale extractions (14).

Other alternatives based on green chemistry have been pursued. Particularly, due to the drawbacks associated with existing processes, the demand for methods that have high extraction yields, low cost, with potential for scale-up production and environmentally friendly persists.

Deep eutectic solvents (DESs) are a new class of green solvents and have received great attention as extraction media. DESs, introduced in the beginning of the 21st century, are prepared by simply mixing at least one hydrogen bond acceptor (HBA) with one hydrogen bond donor (HBD) at an appropriate molar ratio to form a eutectic mixture (15). The strong bonding between HBA and HBD is the most important parameter in the formation of these systems (15). This interaction results in a depression of the melting point of the system relative to its initial components. This simple process of manufacture makes industrial scale production possible without the need for complex facilities and specialized handwork; this method does not need solvents and produces no waste or by-products (16). DESs are, hence, attractive candidates for solvents due to their inherent properties, for example, short preparation time, easy storage, low cost, non-flammability, and high capacity of solvation (15, 17). Besides, DESs have other advantages, including high-thermal and electrochemical stabilities (15).

The majority of DESs proposed so far are based on renewable resources, such as carboxylic and amino acids, sugars, amines, representing a new generation of green solvents (18, 19). These DESs based on natural compounds are known as natural deep eutectic solvents (NADES), resulting in low toxicity and biodegradable solutions (19). Depending on the formulation, some NADES can dissolve natural or synthetic chemicals with different polarities. Additionally, because they are composed of natural metabolites, it makes them theoretically fully biocompatible, being a greener alternative candidate for concepts and applications involving some organic solvents and ionic liquids (19).

To date, numerous articles detailing the use of these solvents as extraction (phenolic acids, flavonoids, and polyphenols) and separation media (dissolving lignocelluloses, separation of an azeotropic mixture, and solid-liquid extraction) are reported in the literature (20–22). The extraction of a solid sample involves the transfer of the analyte to the liquid phase through diffusion or solvation- desorption mechanisms, and the selection of the solvent used depends on the characteristics of the matrix and the analyte. Satisfactory results have been obtained by optimization of the extraction parameters (such as time, sample mass, and extraction solvent volume), in combination with the use of green techniques, such as ultrasound-assisted extraction (UAE).

Publications have already shown the potential of NADES as extraction media to obtain phytocannabinoids (13, 18). However, they do not evaluate the extraction of other bioactive compounds, neither do they evaluate if the solvents affect the bioactivity of the cannabinoids extracted. This work aims to compare the performance of hydrophilic and hydrophobic DES by developing green DESs that allow the effective extraction of cannabinoids and other bioactive agents from *Cannabis sativa* L. and further promote their bioactivity for potential therapeutic applications.

## Materials and Methods

### Chemicals and Reagents

The chemicals used for the preparation of DESs included Betaine (99%), DL-Menthol (≥ 95%), Nile Red (≥ 98%) purchased from Sigma, Lactic acid (85%), Lauric acid (98%), Myristic acid (≥ 98%) obtained from Sigma–Aldrich, L-proline (98%) from Scharlau, D-(+)-glucose anhydrous (≥ 95%), Stearic acid (98%) from Merck/Sigma, Ethanol (96%) from Valente and Ribeiro, Methanol PA from Honeywell, Folin-Ciocalteau Reagent from Panreac, L-Ascorbic acid (99%), Quercetin (HPLC grade), which were purchased from Sigma and were used as purchased.

### Plant Material

*Cannabis sativa L*. (hemp) was provided by South Hemp Tecno srl (Taranto, Puglia), providing dried threshing residues of the EU certificated variety Futura 75. The flowers and leaves were harvested in 2018 from an Italian farm located in Puglia. The material was constituted by a mixture of leaves, flowers, and seeds.

### Sample Preparation

Before extraction, hemp leaves and inflorescences were grinded using a commercial blender. This method allowed a reduction of the sample size and a higher contact between the DES and the plant material, resulting in an increase of the extraction efficiency, consequently, increasing the final yield. The inflorescences, leaves, and seeds were grinded in a lab mill (IKA^®^ Tube-mill control) at 6,000 rpm for 45 s, with a particle size range between 1 mm and 180 μm. The samples were kept in an amber flask in a dark place to protect them from light, under room temperature.

### Deep Eutectic Solvents Preparation

Deep eutectic solvents were produced by the heating-stirring method. This method was selected since it is simple and allows the preparation of multiple DESs simultaneously, and it can be easily scaled up. DESs were obtained by mixing the HBAs and HBDs at the desired molar ratio as shown in Table 1. The mixtures were stirred using a magnetic stirring apparatus at 40°C until a homogenous solution was obtained. All DESs were then stored at room temperature.

**TABLE 1:** An overview of the tested DESs.

No.	HBA	HBD	Abbreviation	Molar ratio
DES 1	Betaine	L(+)-Lactic Acid	Bet:Lac	1:2
DES 2	Glucose	L(+)-Lactic Acid	Lac:Gluc	1:5
DES 3	L-Proline	L(+)-Lactic Acid	Pro:Lac	1:1
DES 4	Menthol	L(+)-Lactic Acid	Men:Lac	2:1
DES 5	Menthol	Lauric Acid	Men:Lau	2:1
DES 6	Menthol	Myristic Acid	Men:MyA	4:1
DES 7	Menthol	Stearic Acid	Men:StA	8:1

### Determination of Physical Properties of the Deep Eutectic Solvents

#### Polarity

Generally, the greater the intermolecular attractions, the larger the polarity. Thus, polarity is generally a solubilization property. To study NADES polarity, solvatochromic studies have been performed using Nile red. Polarity intervals can be identified by referencing against a standard.

$$E_{T}\,\left(N\,R\right)/K\,c\,a\,l.m\,o\,l^{-1}=h\,c\lambda \,\max\text{NA}=28,591/\lambda \,\max,\mathrm{NR}$$

where h is Planck’s constant, c is light speed, and λmax = wavelength of a maximum of UV absorbance.

#### Water Content

After their synthesis, the water content of the DESs was measured using Karl Fischer (Metrohm). For each titration, ≅ 50 mg of the samples was injected. The measurements were made in triplicate.

#### Density and Viscosity

Density, ρ, and viscosity, η, data of the prepared DESs were measured from 293.15 to 343.15 K at atmospheric pressure, with a densimeter/viscosimeter (SVM 3001 from Anton Paar). The temperature was controlled with an accuracy of ± 0.01 K.

### Deep Eutectic Solvents Extraction

Bioactive compounds from hemp samples were extracted by mixing the DES with the plant matrix in a solid-liquid ratio of 1:10 (W/W). After being briefly mixed, cannabinoids were extracted for 90 min in an ultrasonic bath [Model XUB5, Formatura (Type Solution)] (water temperature at 60°C; ultrasonic power, 100 W) in cycles of 15 min; a sample was collected for future kinetic analyses. Then centrifuged (Model Victor Nivo 3S, ILC) at 6,000 rpm for 15 min to separate the liquid from the solid phase. Each experiment was repeated three times for each DES, and the respective cannabinoids were quantified by HPLC.

### Ethanol Extraction

#### Soxhlet Extraction

To compare the efficiency of the DES extraction method, a Soxhlet extraction with ethanol was performed. In this method, 2 g of hemp was extracted with 70 ml of ethanol on a Soxhlet apparatus for 90 min. The resulting solutions were transferred into a weighted flask, the solvent evaporated in a rotatory evaporator and the oils obtained saved at 4°C for future studies. The extractions were carried in triplicate, and the respective cannabinoids were quantified by HPLC analyses.

### Cannabinoids Analysis HPLC

The samples were prepared by diluting the obtained extracts in methanol (a 1:10 w/w ratio) and then stirred to until a homogenous solution was obtained. The solution was then filtered using a hydrophilic PTFE syringe filter with a 0.20-μm pore size (FilterLab) before analysis.

HPLC analysis of the hemp extracts was carried out using an Agilent Infinity 1100 HPLC System, and an Agilent 1100 series photodiode-array detector (DAD) for detection and recording at UV/Vis 220 nm. The cannabinoids chromatographic separations were achieved using a Kinetex C-18 column (100 mm × 4.6 mm ID and 2.6-μm particle size, 100 Å pore size). The method used for the HPLC analysis was adapted from the Cannabinoids on Raptor ARC-18 Restek LC_GN0553 methodology as described elsewhere (23).

As mobile phase A:0.1% Formic acid in water and B:0.1% Formic acid in acetonitrile were used, the solvent flow was kept constant at 1.5 ml/min with the following gradient profile:0.00–4.00 min 25% of solution A, 75% of solution B, 0.00–4.01 min 0% of solution A, 100% B of solution and 4.01–7.00 min 25% of solution A, 75% of solution B. The column oven was kept at 50°C during the run, and the injection volume was of 5 μL. The identification and the quantification of cannabinoids were based on CBD, CBDA external standards.

### Determination of Total Phenolic Content

The total phenolic content (TPC) was determined for individual extracts using the Folin–Ciocalteu method and was adopted from Waterhouse A.L, with some modifications (24). The outcome data were expressed as mg of gallic acid equivalents per gram of hemp (mg GAE/g hemp).

The calibration curve was obtained, preparing a stock solution with concentration of 5 g/L, dissolving 0.5 g of gallic acid in 10 ml of ethanol and 90 ml of distilled water. From this solution, five standard solutions obtained, with concentrations of 50, 100, 150, 250, and 500 mg/L, The five standard solutions obtained, with concentrations of 50, 100, 150, 250, and 500 mg/L, respectively, were used to acquire the calibration standard curve. Briefly, 20 μL of extract (diluted 1:10 w/w in methanol) was mixed with 1.58 ml of distilled water and 100 μL of Folin–Ciocalteu reagent; the solution was mixed and incubated at room temperature for 7 min. Then, 300 μL of a Na_2_CO_3_ solution was subsequently added to the mixture and incubated at 40°C for 30 min. Afterward, the absorbance was measured, utilizing a microplate reader (Model Victor Nivo 3S, ILC) at 750 nm.

### Determination of Total Flavonoids Content (TFC)

The flavonoid content of individual extracts was measured according to Navarro J.M. et al. (25). The calibration curve was prepared from a stock solution with concentration of 2 g/L, dissolving 0.2 g of quercetin in 100 ml of methanol. From this solution, five standard solutions were obtained, with concentrations of 5, 50, 100, 125, and 250 mg/L, respectively, and then used to acquire the calibration standard curve (*R*^2^ = 0.998). Briefly, 125 μL of extract (diluted 1:10 in methanol) was mixed with 37.5 μL of NaNO_2_ (5%) and mixed. After 6 min, 75 μL of AlCl_3_ (10%) was added and incubated for 5 min after mixing. Then, 250 μL of NaOH (1 M) was added. Finally, the mixture was adjusted with 1.25 ml of distilled water. The absorbance versus a prepared blank was read at 510 nm in the microplate reader (Model Victor Nivo 3S, ILC). The total flavonoid content of the samples was expressed as mg of quercetin equivalents per gram of hemp (mg QE/g hemp). The experiments were made in triplicate.

### Extraction of Volatile Compounds (Terpenes) From Hemp by SPME/GC-MS

Terpene’s identification was performed by SPME/GC-MS, using the method described by Stenerson K, with adaptations (26). In this procedure, 1 g of each hemp extract was transferred into a headspace vial. All samples were analyzed using an Equity-1 capillary GC column. Helium was used as carrier gas at 1 ml/min. The inlet temperature was set at 250°C and a split ratio of 1:30. The general extraction procedure was performed with the 100-μm PDMS fiber; the extraction time selected was 30 min during the equilibration time in a thermostatic bath at 40°C with magnetic stirring at 100 rpm. The GC oven temperature gradient started at 45°C, followed by a ramp of 2°C/min to 100°C and another increase from 5°C/min to 250°C. In this study, an analysis of the plant (hemp) was also carried out in order to verify the presence of volatile components, serving later as a means of comparison with the extractions performed with the three systems.

### Wax Quantification

The removal and the quantification of waxes were carried out through a process called “winterization” of oil and were applied to the obtained extracts. The analysis was carried out in triplicate. Briefly, 1–3 g of extract was dissolved in 10–30 ml of ethanol (96%), respectively, and stirred until a homogeneous solution was obtained. The solution was then left to cool down in the freezer (-20°C) to induce wax precipitation for 24 h. The samples were then centrifugated at 6,000 rpm for 15 min to separate the waxes from the solution. The solid residue was then left to evaporate the remaining ethanol overnight and then weighted to calculate the wax concentration (% W/W). The supernatant was then transferred for a volumetric balloon to evaporate the solvent by a rotary evaporator. The recovered dewaxed extracts were kept at 4°C and in darkness until analysis.

$$Waxquantification\left(\%\right)=\frac{E\,x\,t\,r\,a\,c\,t\,e\,d\,w\,a\,x\,e\,s\,\left(g\right)}{F\,e\,e\,d\,m\,a\,s\,s\,\left(g\right)}\times 100$$

### Total Chlorophyll Quantification

Chlorophyll (CHL) content was determined by following the method previously described by Arnon (27). Briefly, approximately 0.1 g of extract was diluted in 10 ml of ethanol, 96% (v/v). After dilution, samples were centrifuged (6,000 rpm for 15 min) and the absorbance of the supernatants measured at 663 and 645 nm. Contents of CHL a, CHL b, and total CHL were calculated as follows:

C⁢H⁢L⁢a=⁢(12, 25×A⁢b⁢s⁢o⁢r⁢b⁢a⁢n⁢c⁢e⁢a⁢t⁢ 663⁢nm)-(2, 79×A⁢b⁢s⁢o⁢r⁢b⁢a⁢n⁢c⁢e⁢a⁢t⁢ 645⁢nm)

C⁢H⁢L⁢b=⁢(21, 50×A⁢b⁢s⁢o⁢r⁢b⁢a⁢n⁢c⁢e⁢a⁢t⁢ 645⁢nm)-(5, 10×A⁢b⁢s⁢o⁢r⁢b⁢a⁢n⁢c⁢e⁢a⁢t⁢ 663⁢nm) T⁢o⁢t⁢a⁢l⁢C⁢H⁢L=C⁢H⁢L⁢a+C⁢H⁢L⁢b

Results of three measurements were expressed as μg total CHL/g extract.

### Antioxidant Activity Evaluation – 1,1-Diphenyl-2-Picrylhydrazyl

The antioxidant activity of the extracts was measured based on their scavenging activity of the stable 1,1-diphenyl-2-picrylhydrazyl (DPPH) free radical. About 150 μL of the extracts was added to 4 ml of the DPPH working solution. After incubating for 40 min at room temperature, the absorbance of the preparations was taken at 517 nm by a microplate reader (Model Victor Nivo 3S, ILC). Sample antioxidant activity was compared to standard ascorbic acid concentrations (1–500 μg/ml). Then, the % inhibition was calculated by the following equation:

$$RSA\left(\%\right)=\frac{\left(A\,b\,s\,o\,r\,b\,a\,n\,c\,e\,o\,f\,b\,l\,a\,n\,k-A\,b\,s\,o\,r\,b\,a\,n\,c\,e\,o\,f\,s\,a\,m\,p\,l\,e\right)}{A\,b\,s\,o\,r\,b\,a\,n\,c\,e\,o\,f\,b\,l\,a\,n\,k}\times 100$$

From the calibration curves, determined from different concentrations of the extracts, the IC50 was obtained. IC50 value denotes the concentration of the sample required to scavenge 50% of the DPPH free radicals.

### Solubility Measurements

The solubility of CBD and CBDA in PBS was determined *via* the shake-flask method, which consisted in dispersing an excessive amount of extract in 2 ml of phosphate-buffered saline solution (PBS Sigma Aldrich, St. Louis, MO, United States) and stirred at 300 rpm, for 24 h, at 37°C in a water bath. When the assay ended, the samples were centrifuged (Model Z 206, Hermie) at 6,000 rpm for 15 min, and the supernatant was collected. The obtained samples were filtered, using a hydrophilic PTFE syringe filter with a 0.22-μm pore size (Filter Lab, Barcelona, Spain) and analyzed by HPLC. The solubility measurements were carried out in triplicate to determine the saturation concentration of CBD + CBDA in the buffer.

### Statistical Analysis

The results of cannabinoids, wax, and chlorophyll extraction where all statistically treated with Graph Pad Prism 6. To indicate statistically significant differences between means, the mean value obtained from each DESs tested was compared to the control using one-way ANOVA and a confidence interval of 95% (*p* = 0.05). When the standard deviations of the group presented significant differences (*p* < 0.05), the Turkey multiple comparison test was performed to compare each mean value to the different solvents.

## Results and Discussion

### Deep Eutectic Solvents Preparation

Seven DESs were successfully prepared as homogenous liquids, without any crystal precipitation at normal ambient temperature, excluding for Men:StA, which is solid at room temperature. All the systems appear as a transparent liquid except for Pro:Lac, which is a yellowish liquid.

### Physico-Chemical Characterization of the Deep Eutectic Solvents Prepared

The determination of the physico-chemical properties of DESs is extremely important since they have a significant influence on the solvent properties, affecting solvents suitability for specific applications. Thus, polarity, water content, density, and viscosity of the prepared systems were determined.

Experimental data on polarity obtained using Nile red as a probe and the water content measured by the Karl Fischer titrator are shown in Table 2.

**TABLE 2:** Experimental water content (%) and polarity (E_*T*_NR) of the DESs:Betaine lactic acid Bet:Lac (1:2), lactic acid glucose Lac:Gluc (5:1), proline lactic acid Pro:Lac (1:1), menthol lactic acid Men:Lac (2:1), menthol lauric acid (2:1), menthol myristic acid Men:MyA (4:1), and menthol stearic acid.

DESs	Water Content (%)	E_*T*_NR(Kcal/mol)
Bet:Lac	6.7 ± 0.8	50.16
Lac:Gluc	9.0 ± 0.6	44.67
Pro:Lac	4.5 ± 0.5	49.58
Men:Lac	2.4 ± 0.1	51.80
Men:Lau	0.1 ± 0.01	53.34
Men:MyA	0.1 ± 0.02	53.34
Men:StA	0.2 ± 0.02	–

*Composition of DES is expressed in molar ratios.*

Nile red is a molecule whose florescence is influenced by the polarity of where it is dissolved. The polarity evaluation of Men:StA could not be assessed since this measurement is performed at room temperature at which the solvent is in the solid state, making it impossible to read on the spectrophotometer. The higher the E_*T*_NR value, the more non-polar the solvent is. The polarity values in Table 2 allowed us to distinguish the prepared DESs in two groups: hydrophilic (Low E_*T*_NR) and hydrophobic (High E_*T*_NR). Comparing the results, Lac:Gluc is the most polar solvent, followed by Pro:Lac, Bet:Lac, Men:Lac, Men:Lau, and Men:MyA, the least polar. These values are in consistence with the amount of water present in the solvents. Men:Lac presented the highest water content of all DESs; this is easily explained by the use of Lactic acid (85%) in the composition of the system, which rises the water present in the solvent, decreasing E_*T*_NR, making it slightly more polar than the other hydrophobic DESs.

The determination of density and viscosity was carried out at atmospheric pressure from 293.15 to 338.15 K. Due to their high viscosity, it has not been possible to determine the viscosity in the whole range of temperatures for all of them. Results are shown in Tables 3, 4. The extraction efficiency is substantially influenced by the level of solvent penetration into the biomass structure; the lower the solvent density is, the more effective is its penetration into the raw material’s matrix (28). Experimental density values listed in Table 3 showed that, for all the tested systems, the increase in temperature resulted in the decrease of density. The amino acid-based DESs presented the highest density values, while menthol-based DESs presented the lowest. At 60°C, temperature at which extraction was performed Lac:Gluc presented the highest density, followed by Pro:Lac, Bet:Lac, Men:Lac, Men:Lau, and Men:StA as the lowest. From the viewpoint of the efficiency of penetration into the biomass, it can be supposed that Lac:Gluc would be the least effective.

**TABLE 3:** Experimental densities, ρ, expressed in g/cm^3^, of the DESs:Betaine lactic acid Bet:Lac (1:2), lactic acid glucose Lac:Gluc (5:1), proline lactic acid Pro:Lac (1:1), menthol lactic acid Men:Lac (2:1), menthol lauric acid (2:1), menthol myristic acid Men:MyA (4:1), and menthol stearic acid (8:1).

T/K	Bet:Lac (1:2)	Lac:Gluc (5:1)	Pro:Lac (1:1)	Men:Lac (2:1)	Men:Lau (2:1)	Men:MyA (4:1)	Men:StA (8:1)
293.15	1.20	1.28	1.27	0.95	0.90	0.90	
298.15	1.20		1.26		0.89		
303.15	1.19	1.28	1.26	0.95	0.89	0.89	
308.15	1.19		1.26		0.89		
313.15	1.19	1.27	1.25	0.94	0.88	0.88	0.88
318.15	1.19		1.24		0.88		0.88
323.15	1.18	1.26	1.24	0.93	0.87	0.87	0.87
328.15	1.18		1.24		0.87		0.87
333.15	1.18	1.25	1.24	0.92	0.87	0.87	0.87
338.15	1.17		1.23				0.86
343.15	1.17		1.23		0.86		0.86

*Composition of DES is expressed in a molar ratio.*

**TABLE 4:** Experimental viscosities, η, expressed in mPA/s, of the DESs:Betaine lactic acid Bet:Lac (1:2), lactic acid glucose Lac:Gluc (5:1), proline lactic acid Pro:Lac (1:1), menthol lactic acid Men:Lac (2:1), menthol lauric acid (2:1), menthol myristic acid Men:MyA (4:1).

T/K	Bet:Lac (1:2)	Lac:Gluc (5:1)	Pro:Lac (1:1)	Men:Lac (2:1)	Men:Lau (2:1)	Men:MyA (4:1)	Men:StA (8:1)
293.15		818.73		85.25		46.32	
298.15	1266				32,03		
303.15	819	330.73		38.77	18.46	24.25	
308.15	545				14.39		
313.15	375	152.88		20.30	11.42	13.96	16.61
318.00	261				9.21		12.62
323.15	190	79.30	997.4	11.85	7.54	8.71	9.81
328.15	142		675.1		6.25		7.77
333.15	108	45.18	472.4	7.56	5.25	5.82	6.27
338.15	84		338.9		4.46		5.14
343.15	66		246.1		3.83		4.27

*Composition of DES is expressed in molar ratios.*

The high viscosity of DESs can make them difficult to handle in industrial processes during processing and filtration, even though it sharply decreases when temperature increases (28). Experimental viscosity data, reported in Table 4, showed that, with an increase of temperature, a decrease on viscosity was observed. The hydrophilic DESs presented the highest viscosity, being Pro:Lac the more viscous of all systems. At 60°C, Pro:Lac presented the highest viscosity, followed by Pro:Lac, Bet:Lac, Men:Lac, and Men:Lau, as the lowest. These high values at this temperature could result in a less-effective extraction process due to problems in mass transfer and, therefore, a lower extraction yield.

### Cannabinoid Extraction From Hemp

#### Effects of Deep Eutectic Solvent Composition on Extraction

**FIGURE 1:**
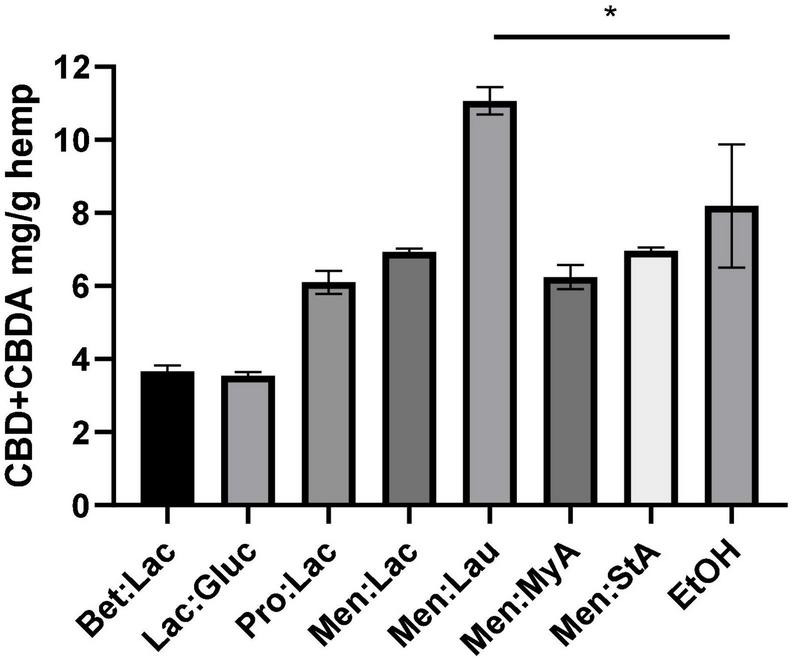
Extraction of CBD + CBDA from the hemp using different DESs. The extraction conditions were as follows: 60°C, 90 min, and a 1:10 solid-liquid ratio (w/w). The symbol * means that there are statistical differences between Men:Lau and EtOH.

The choice of the solvent, in this case the DES, is one of the most important things in solid-liquid extraction. Therefore, seven different DESs were chosen as potential candidates. The screening was carried out, fixing a 1:10 solid-liquid ratio for the extraction of bioactive compounds from *Cannabis sativa* L. The results of the extraction yield using different solvents are represented in Figure 1. The extraction efficiency is herein assessed through the quantification of CBD and CBDA in the extract as mg CBD + CBDA/g hemp.

As it can be seen, the extraction solvent used has a significant effect on the extraction efficiency. The extraction yield decreased with the use of hydrophilic DESs (Bet:Lac, Lac:Gluc, and Pro:Lac). Menthol-based hydrophobic system produced the highest yields of all DESs that were tested (Men:Lac, Men:Lau, Men:MyA, and Men:StA). In summary, the best result for the hydrophobic DES was obtained for Men:lau (11.07 ± 0.4 mg/g), and, for the hydrophilic ones, Pro:lac (6.1 ± 0.3 mg/g) was the best solvent. These results indicate that the interactions between DESs and target compounds affect the extraction ability of DESs. This effect could be accounted for two reasons: among other characteristics, polarity is one of the most important properties, and it is a key indicator of the DESs dissolving capability (13). Hydrophilic systems are polar solvents, while cannabinoids are non-polar molecules; these differences in polarity result in fewer interactions between the solvent and the target compounds, resulting in a poor extractability and subsequent less-concentrated extracts. In the case of hydrophobic systems, the similar polarities allow a higher affinity with the target compounds, resulting in a greater extraction yield. Another reason is the viscosity of the systems; hydrophilic systems are more viscous than menthol-based hydrophobic systems. The increase of viscosity influence on the effectiveness of the separation process is due to the decrease of the mass transfer (29).

In this work, a soxhlet extraction with ethanol was performed both to characterize the plant and compare the extraction efficacy of this solvent with the tested DESs under the same extraction conditions. The extraction efficiency of Men-Lau (11.07 ± 0.4 mg/g) has demonstrated to exceed the performance of ethanol (8.19 ± 1.7 mg/g).

The statistical analysis of the extraction performance showed statistically significant differences between Men:Lau and ethanol (one-way ANOVA, *p* < 0.05). Turkeys multiple comparisons analysis did not show significant differences between ethanol and Men:Lac, Men:MyA, and Men:StA, (*p* > 0.05). These results demonstrate that hydrophobic DESs are equally effective as ethanol. Based on these results, Men:Lau, with the greatest overall extraction efficiency, should be selected for the extraction of CBD and CBDA from hemp.

Vági E. et al. (30) showed the effect of supercritical CO_2_ on the extraction of cannabinoids from hemp. The extracts obtained contained 1.63–7.88 mg/g d.m. of CBD and 1.01–16.60 mg/g d.m. of CBDA (30). Our extraction yields using DESs are between these values, proving the efficiency of this extraction method. It is also important to notice that these extractions with DESs were carried out without further optimization.

#### Effect of Extraction Time

**FIGURE 2:**
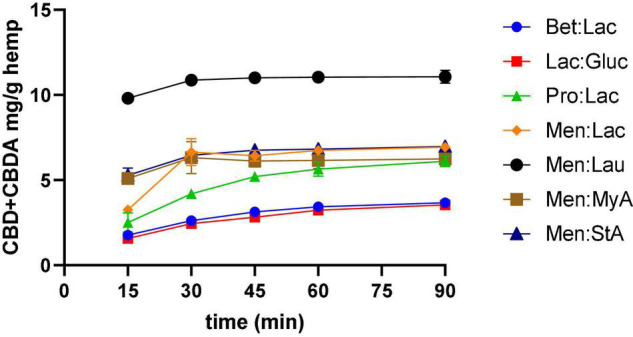
Effect of time in CBD + CBDA extraction. Extraction conditions were 60°C, a 1:10 solid-liquid ratio.

To represent the kinetic curves of the process, samples were collected along the extraction every 15 min, and the results are illustrated in Figure 2 and refer to all the DESs extractions performed. The kinetic curves allowed to understand the efficacy of the extraction through time and optimize the processing time as the extractions need to be short to decrease costs. The results showed that, for Bet:Lac, Lac:Gluc, and Pro:Lac, the extraction yield increased from 15 to 60 min, and then varied little with time. Similar results were observed by Changyong Cai et al. (13). Therefore, the extraction time should be 60 min. For Men:Lac, Men:Lau, Men:MyA, and Men:StA, the extraction yield varied little after 30 min of extraction. Therefore, the extraction time, in this case, should be 45 min. These systems allow a higher extraction yield in a shorter time frame, being preferable for industrial applications.

### Chemical Characterization of the Hemp Extracts

The characterization of the extracts included not only the evaluation of the content in CBD and CBDA but also the quantification of other bioactive compounds, such as the TPC, total flavonoid content, and terpene content. The presence of subproducts of extraction was also evaluated, such as wax and total chlorophyll, in order to evaluate the specificity of the extraction solvents.

#### Total Phenolic Content

**FIGURE 3:**
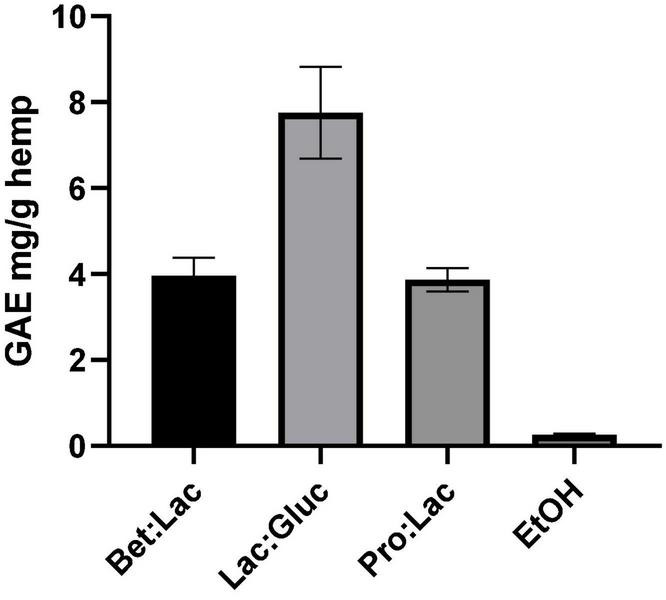
Total phenolic content related to the different solvents tested and expressed in mg GAE/g hemp (extraction conditions: 60°C, 90 min, and a 1:10 S/L ratio).

Phenolic compounds are important plant constituents with redox properties responsible for antioxidant activity, and their extraction requires compatible solvents with high commercial interest. The results obtained with the Folin-Ciocalteu method allowed to quantify the TPC using the gallic acid standard as equivalent. The extraction efficiency is herein assessed through the quantification of TPC in the extract as mg GAE/g hemp. The values are derived from the calibration curve and are illustrated in Figure 3 in relation to the solvents used. The TPC in the hydrophobic DESs was not possible to assess due to the incompatibility of the method with the presence of menthol as the protocol used led to the formation of a precipitate, making it impossible to get an accurate measurement. However, since phenolic compounds are polar molecules, their extraction yield with the menthol-based DESs is expected to be very low. As it can be seen, the solvent used affected the extraction of TPC. Among all the tested DESs, the best performance was observed for Lac:Gluc (7.76 ± 1.1 mg GAE/g). From the results reported, it can be assumed that an increase of the polarity of the solvent has a positive effect on the TPC, as the highest value was obtained for Lac:Gluc, which has the highest polarity and the higher water content. These results are in accordance with the literature. The phenolic compounds possess higher polarity when compared with cannabinoids and terpenes due the higher ratio oxygen/carbon. These differences in polarity make the phenolic fraction less soluble into ethanol, which, instead, is non-polar. As a result, the phenolic compounds are only partially extracted with ethanol (0.26 ± 0.02 mg GAE/g), which is richer in non-polar compounds, such as cannabinoids (31). Lac:Gluc also presented the lowest viscosity of all systems; this factor could also facilitate the mass transfer phenomena and improve the extraction yield.

The results also demonstrate that we can have different selectivities for TPC according to the composition of the DESs. Lac:Gluc extraction conditions could also be optimized for a higher extraction yield.

#### Total Flavonoid Content

**FIGURE 4:**
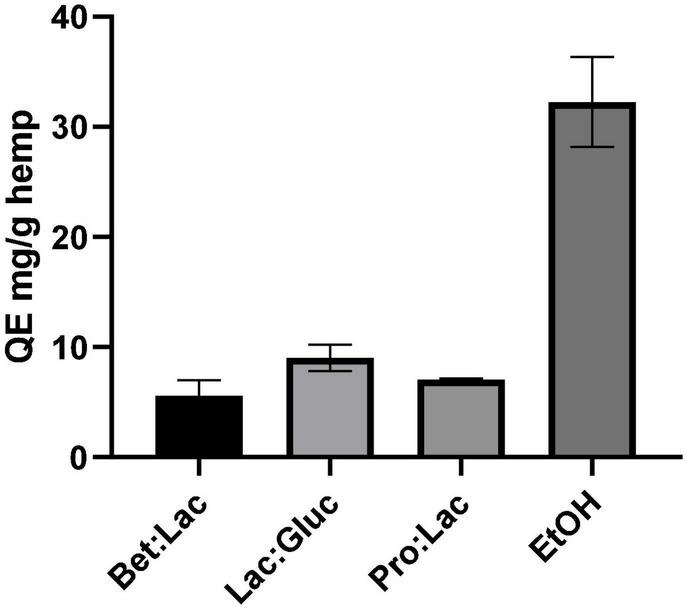
Total flavonoid content related to the different solvents tested and expressed in mg QE/g hemp (extraction conditions: 60°C, 90 min, and a 1:10 S/L ratio).

The total flavonoids content (TFC) was determined following the aluminum colorimetric method, which allowed to quantify these compounds using the quercetin standard as equivalent. The results were derived from the calibration curve of quercetin and expressed in mg QE/g hemp and are illustrated in Figure 4 in relation to the solvents used. Once again, the TFC of the hydrophobic DESs could not be determined due to the incompatibility of the method with the presence of menthol. As seen for the TPC, the solvent used affects the TFC extraction yield. Among all the tested DESs, the best performance was observed once again for Lac:Gluc (9.02 ± 1.2 mg QE/g); however, the efficacy of extraction was exceeded by ethanol (32.25 ± 4.1 mg QE/g). From these results, it can be assumed that an increase of the E_*T*_NR polarity of the solvent, on the contrary, of the total phenolics has a negative effect on the TFC as the highest value was obtained for EtOH (32.25 ± 4.1 mg QE/g extract), which is the less polar of the tested solvents. This high value is explained since the majority of phenolic compounds present in cannabis are flavonoids. Among all DESs, no differences in performance were observed in TFC extraction.

#### Terpene Extraction

Cannabis is composed of a wide variety of terpenes that provide therapeutic benefits and properties (32). The results obtained through the combination of SPME and GC/MS allowed the qualitative analysis through identification of the terpenes composition on the hemp extracts obtained with hydrophilic DESs and are presented in Table 5. This technique could not be used for the identification of terpenes in the hydrophobic extracts. As menthol is a terpene and is in a high concentration in the hydrophobic DESs, this would result in the saturation of the fiber overlapping the peaks of the additional terpenes that could be present in the extracts. Besides the identification of the terpenes present in the extracts, we also performed an analysis of the plant matrix used as control.

**TABLE 5:** Identification of the terpenes present in DESs extracts by GC-MS/SPME.

DES	α -pinene	β -pinene	β -myrcene	β -limonene	*Trans-*caryophyllene	α -cariophyllene	Caryophyllene oxide	β -Selinene
Bet:Lac	✓	–	✓	✓	✓	✓	–	✓
Lac:Gluc	✓	–	✓	✓	✓	✓	–	✓
Pro:Lac	✓	✓	✓	✓	✓	✓	✓	✓
Hemp	✓	✓	✓	✓	✓	✓	–	✓

From the data presented, it was possible to observe that all the tested DESs can extract the main terpenes present in hemp. It was also visible that the main fraction of terpenes present belongs to the monoterpenes (α-Pinene, β-Pinene, β-Myrcene, and β-Limonene) and to the sesquiterpenes group (*Trans-*Caryophyllene, α-Caryophyllene, Caryophyllene oxide, and β-Selinene). These results are in accordance with the typical terpenes composition in terms of the qualitative analysis found in cannabis (32). All the terpenes found in the extracts were also detected in the analysis of the plant matrix, except caryophyllene oxide. This result can be due to the fact that this terpene is less volatile and not extracted just by heating and stirring of the plant in the headspace.

#### Wax Quantification

**FIGURE 5:**
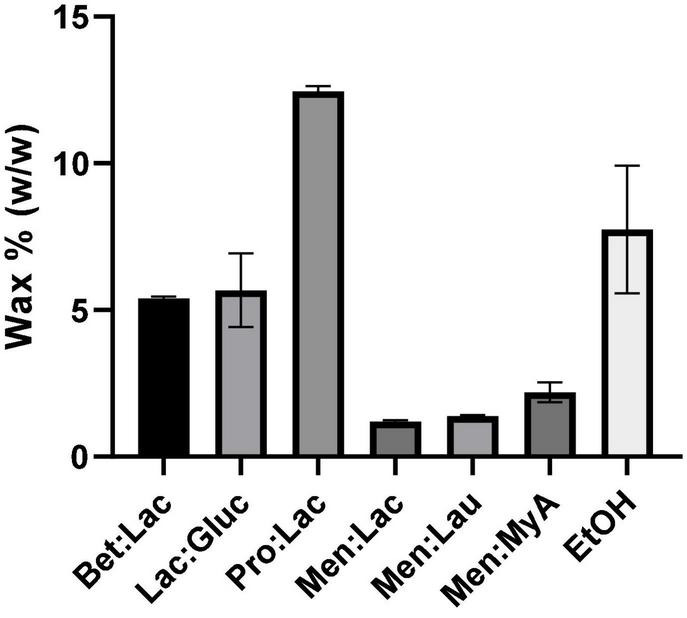
Results of wax quantification (%) of hemp extracts related to the different extraction solvents.

During the extraction process, some compounds are extracted alongside the targeted compounds, such as waxes, as a result of unselective extraction media. The cannabis wax layer is easily soluble in many solvents that are used for extraction, such as supercritical CO_2_ and organic solvents, such as ethanol. While waxes are useful for plants, they are often an undesirable by-product of extraction, since they decrease the purity of the extracts and increase the overall cost of extraction with the addition of purification steps (33). In this work, we evaluated the wax extraction capability of all DESs tested and compared with ethanol. The results obtained from the wax quantification (%) are presented in Figure 5. Men:StA extracts could not be used for the waxes quantification, since, at the winterization temperature, the extracts became solid, making it impracticable for waxes to precipitate.

The results showed once again a significant difference between hydrophobic and hydrophilic DESs. Hydrophilic DESs extracted more waxes, being the highest value obtained for Pro:Lac (12.46% ± 0.2), while hydrophobic extracted the lowest, being Men:Lac the DES, which extracted the least (1.19% ± 0.05). Ethanol showed a behavior very similar to the hydrophilic DESs, having the second highest extraction value (7.75% ± 2.2). These results can strongly be correlated with the polarity of the solvents, where a higher polarity can lead to a higher interaction of the solvent with the waxes, increasing the solubility and leading to a higher extraction. Menthol-based DESs showed to be more specific as extraction media, being a better choice than ethanol, for purer extracts.

#### Total Chlorophyll Extraction

Chlorophylls are natural-occurring pigments present in all plants. During cannabinoids extraction, these subproducts are also extracted from the plant matrix, and there is the need to be removed through purification processes, increasing the overall cost of extraction. Even though some publications have reported the therapeutic benefits of CHL, in an extract that is wanted to be as pure as possible, their presence may be seen as unfavorable (34).

**FIGURE 6:**
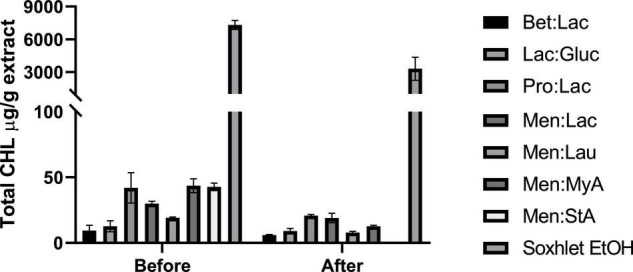
Quantification of the total chlorophyll (μg/g extract) present in the obtained hemp extracts before and after winterization.

In this study, we evaluated the total CHL present in the extracts before and after winterization; the results are expressed in CHL mg/g extract and represented in Figure 6. Ethanol is highly effective in chlorophyll extraction, and this trend was also observed by Sartory and Grobbelaar (35), where they showed that ethanol is more effective in CHL extraction than acetone. Overall, DESs are much more ineffective in chlorophyll extraction when compared to ethanol, showing its selectivity in the extraction process. Menthol-based DESs extracted the highest amount of CHL among all DESs tested. The low polarity of these systems makes them able to extract more fat-soluble molecules, such as CHL. Bet:Lac and Lac:Gluc performed the best in reducing the amount of chlorophyll in the extracts; although they extracted the lowest amount of cannabinoids, they would be preferable to obtain extracts with a low-CHL content. After winterization, it was noticed that there was a significant reduction in the total CHL present in each extract.

### Evaluation of the Bioactivity of the Hemp Extracts

In this work, besides the evaluation of the potential of DESs as extractants, we also studied if their presence in the final product could potentiate the bioactivity of the extracts since many articles have claimed the use of DESs for the solubilization of poorly water-soluble drugs and transdermal drug delivery. In that way, antioxidant activity and solubility were evaluated.

#### 1,1-Diphenyl-2-Picrylhydrazyl Radical Scavenging Activity

Antioxidants are extremely important substances that possess the ability to protect the body from damage caused by free radical-induced oxidative stress (36). The antioxidant potential of the obtained extracts was determined by DPPH before and after winterization, and compared with reference antioxidant ascorbic acid (vitamin C). Also, to evaluate if the solvent (pure DESs) used in the extraction could potentially influence the antioxidant measurements, since it is present in the final extracts, its radical scavenging activity was also evaluated using the same method. The results are presented in Table 6.

**TABLE 6:** The IC50 values of DPPH scavenging effect of hemp extracts (mg/L).

	IC50 mg/L	
	Before winterization	After winterization
Bet:Lac	337.3 ± 37.2	337.3 ± 37.8
Lac:Gluc	226.7 ± 16.4	337 ± 4.5
Pro:Lac	120.2 ± 10.1	112 ± 9.8
Men:Lac	626.8 ± 19.9	696.7 ± 140.4
Men:Lau	670.6 ± 37.1	851.1 ± 344
Men:MyA	773. ± 122.3	792.3 ± 30.1
Men:StA	1689.6 ± 167.1	–
Ascorbic Acid	104.5 ± 4.9	

All the hemp extracts before and after winterization showed concentration-dependent increase in radical scavenging capacity. Among all the samples before winterization, the greatest DPPH radical scavenging potency was recorded for Pro:Lac, followed by the systems Lac:Glu and Bet:Lac. The free-radical scavenging activity of Men:Lac, Men:Lau, Men:MyA, and Men:StA was very weak and did not increase much as the concentration increased. Comparing the extracts with ascorbic acid, which was used as control, the DES Pro:Lac has shown similar IC50 values. The analysis of DPPH scavenging activity results indicated that sugar and amino acid-based DES were the most effective DPPH radical scavengers, explained by the presence of bioactive compounds, such as phenolic compounds, in the extracts, which have a significant antioxidant activity. Even though Pro:Lac did not have the highest TPC and TFC content, they can be richer in other bioactives with antioxidant activity, such as terpenes, which could explain their low IC50; in this work, the quantitative analysis of these compounds was not performed (37). After winterization, the IC50 increased, which could be related to the loss of antioxidant compounds during winterization.

The results of radical scavenging activity of the pure DESs showed that DESs have very little effect on the DPPH assay results, providing evidence that the effects are due to the bioactive compounds extracted.

### Solubility Studies

**FIGURE 7:**
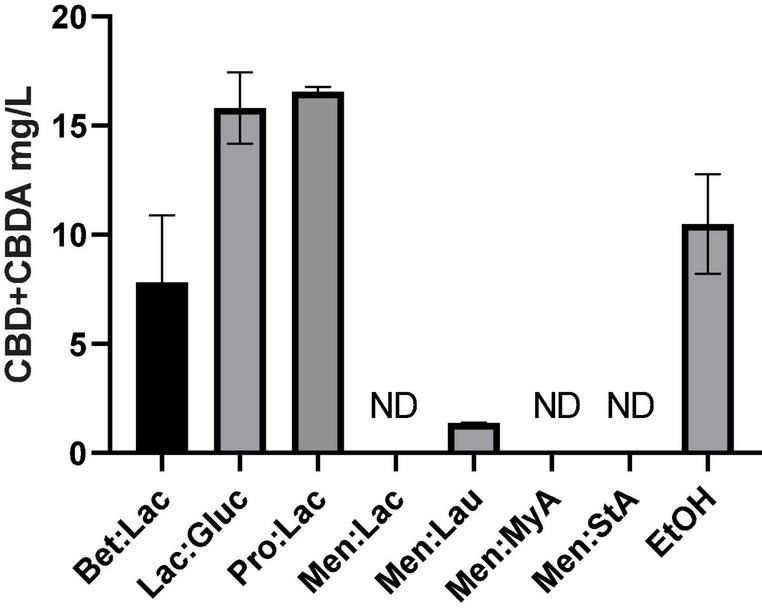
Solubility of CBD + CBDA (mg/L) in PBS at 37°C.

Poor aqueous solubility of the terpenophenolic compound cannabidiol (CBD) is a major issue in the widespread use of this therapeutic molecule (38). As previously described, cannabinoids are highly lipophilic, resulting in very low solubility in water, which has been described in the literature to range between 2 and 10 μg/ml (39). Consequently, these compounds are not readily absorbed, and, therefore, a large dosage is required to have medical effect, presenting a major problem for product design and formulation, besides the costs associated. The development of aqueous solubility-enhanced formulations may lead to higher CBD absorption. DESs comprising or acting as solvents of active pharmaceutical ingredients (API-DESs) have been used to design polymeric drug delivery system, overcome polymorphism, enhance a dissolution rate, increase membrane permeability, and improve transdermal delivery (16). In Figure 7, the solubility values of CBD and CBDA in PBS at 37°C to simulate the physiologic conditions using the tested DESs are represented. Hydrophilic DESs present a higher CBD + CBDA solubility in PBS when compared to cannabis oil and hydrophobic DESs. Lac:Gluc and Pro:Lac presented the highest solubility of all tested DESs. These systems are capable of dissolving in PBS, acting like a carrier for cannabinoids and increasing its solubility. Hydrophobic systems, on the other hand, did not improve the solubilization of cannabinoids. Cannabinoids generally have good solubility in triglyceride lipid bases, allowing for easy solubilization in lipid formulations; since they are more stable in this phase, they do not transfer to PBS.

The statistical analysis of the solubility values showed statistically significant differences between the different solvents (one-way ANOVA, *p* < 0.05); Lac:Gluc and Pro:Lac showed to be statistically different from EtOH (Turkey’s multiple comparisons test, *p* < 0.05). These results demonstrate that these two systems can, in fact, enhance the solubilization of CBD and CBDA in PBS.

## Conclusion

In this study, seven DESs were developed as greener solution media for the UAE of cannabinoids and other main bioactive compounds from hemp. Moreover, DESs were evaluated not only as solvents but also as agents to improve the bioactivity of the target compounds.

The first extraction screening in hemp showed that menthol-based hydrophobic DESs showed to be more efficient in the extraction of cannabinoids. Hydrophilic systems presented low cannabinoid extractability; however, they were able to extract important antioxidant compounds, such as phenolic compounds, flavonoids, and terpenes. Lac:Gluc had the highest TPC values (7.76 ± 1.1 mg/g) and TFC among all DESs. When comparing the extraction results with ethanol, Men:Lau was the one who presented the highest yield of CBD and CBDA (11.07 ± 0.4 mg/g). These systems also proved to be more selective, reducing the extraction of undesirable compounds, such as chlorophyll and waxes.

Bioactivity assays showed that Lac:Gluc and Pro:Lac also improve the solubility of CBD and CBDA in aqueous media. Therefore, the results of this study prove that DESs are selective green solvents, with huge potential for use in industrial applications, involving the extraction of bioactive compounds, and can further enhance the bioavailability of the active components.

## Data Availability Statement

The original contributions presented in the study are included in the article/supplementary material, further inquiries can be directed to the corresponding author/s.

## Author Contributions

FT was responsible for all the laboratory work. AP, AM, and AD were responsible for supervising and review the results. All authors contributed to the article and approved the submitted version.

## Conflict of Interest

The authors declare that the research was conducted in the absence of any commercial or financial relationships that could be construed as a potential conflict of interest.

## Publisher’s Note

All claims expressed in this article are solely those of the authors and do not necessarily represent those of their affiliated organizations, or those of the publisher, the editors and the reviewers. Any product that may be evaluated in this article, or claim that may be made by its manufacturer, is not guaranteed or endorsed by the publisher.
